# Signal processing by T-type calcium channel interactions in the cerebellum

**DOI:** 10.3389/fncel.2013.00230

**Published:** 2013-11-27

**Authors:** Jordan D. T. Engbers, Dustin Anderson, Gerald W. Zamponi, Ray W. Turner

**Affiliations:** ^1^Department of Cell Biology and Anatomy, Hotchkiss Brain Institute, University of CalgaryCalgary, Canada; ^2^Department of Physiology and Pharmacology, Hotchkiss Brain Institute, University of CalgaryCalgary, Canada

**Keywords:** Cav3, HCN, T-type, IKCa, KCa3.1, channel complex

## Abstract

T-type calcium channels of the Cav3 family are unique among voltage-gated calcium channels due to their low activation voltage, rapid inactivation, and small single channel conductance. These special properties allow Cav3 calcium channels to regulate neuronal processing in the subthreshold voltage range. Here, we review two different subthreshold ion channel interactions involving Cav3 channels and explore the ability of these interactions to expand the functional roles of Cav3 channels. In cerebellar Purkinje cells, Cav3 and intermediate conductance calcium-activated potassium (IKCa) channels form a novel complex which creates a low voltage-activated, transient outward current capable of suppressing temporal summation of excitatory postsynaptic potentials (EPSPs). In large diameter neurons of the deep cerebellar nuclei, Cav3-mediated calcium current (*I*_T_) and hyperpolarization-activated cation current (*I*_H_) are activated during trains of inhibitory postsynaptic potentials. These currents have distinct, and yet synergistic, roles in the subthreshold domain with *I*_T_ generating a rebound burst and *I*_H_ controlling first spike latency and rebound spike precision. However, by shortening the membrane time constant the membrane returns towards resting value at a faster rate, allowing *I*_H_ to increase the efficacy of *I*_T_ and increase the range of burst frequencies that can be generated. The net effect of Cav3 channels thus depends on the channels with which they are paired. When expressed in a complex with a K_Ca_ channel, Cav3 channels reduce excitability when processing excitatory inputs. If functionally coupled with an HCN channel, the depolarizing effect of Cav3 channels is accentuated, allowing for efficient inversion of inhibitory inputs to generate a rebound burst output. Therefore, signal processing relies not only on the activity of individual subtypes of channels but also on complex interactions between ion channels whether based on a physical complex or by indirect effects on membrane properties.

## INTRODUCTION

Voltage- or calcium-gated ion channels can alter the output of a postsynaptic cell by modulating temporal or spatial summation of synaptic responses and thus the ability to attain spike threshold. Since the majority of a synaptic potential is subthreshold to spike generation, ion channels must be activated within a low voltage range or even during membrane hyperpolarizations. A limited subset of ion channels have the requisite properties to function in this manner, with low voltage-activated Cav3 T-type calcium channels directly activated during subthreshold postsynaptic potentials, and HCN channels activated within the negative voltage range traversed by inhibitory postsynaptic potentials (IPSPs; Magee and Johnston, 1995; Magee et al., 1995; Luthi and McCormick, 1998b; Swensen and Bean, 2003; Kole et al., 2006; Hildebrand et al., 2009; Engbers et al., 2011; Gastrein et al., 2011; Engbers et al., 2012). However, the ability for these channels to regulate synaptic responses can differ depending on direct or indirect interactions with other ion channels.

Interactions between calcium and potassium channels can be detected simply at a functional level, in which calcium influx is shown to drive potassium channel activation, or as an actual association at the molecular level based on either a direct interaction between alpha subunits or indirectly through an accessory protein. Cav3 channels have been shown to at least functionally couple to small conductance (SK, KCa2.x) calcium-dependent potassium channels (Wolfart et al., 2001; Wolfart and Roeper, 2002; Yanovsky et al., 2005; Cueni et al., 2008) or big conductance (BK, KCa1.1) potassium channels (Smith et al., 2002). More recently Cav3 channels have been found to associate at the molecular level with a host of potassium channels, including BK channels (Engbers et al., 2013; Rehak et al., 2013), intermediate conductance calcium-dependent potassium channels (IKCa, KCa3.1; Engbers et al., 2012), and the voltage-gated Kv4 family (Anderson et al., 2010a, b). Interactions between these channels allow Cav3-mediated calcium influx to modulate spike repolarization (Gittis and du Lac, 2007; Rehak et al., 2013), firing rate gain (Smith et al., 2002; Anderson et al., 2010a, 2013), and temporal summation of EPSPs (Engbers et al., 2012), all over a wider range of membrane voltage than would be possible with high voltage-activated (HVA) calcium channels. The roles for Cav3 calcium influx in modulating subthreshold responses can also reflect a synergistic interplay with other channels through their respective effects on membrane potential, such as the well-recognized case of Cav3 calcium and HCN channels that drive subthreshold oscillations in thalamic neurons (McCormick and Pape, 1990; McCormick and Bal, 1997; Kim et al., 2001; Cheong and Shin, 2013). HCN channels that give rise to *I*_H_ are also recognized to modulate temporal summation of both excitatory (Magee, 1999; Angelo et al., 2007; Tsay et al., 2007) and inhibitory (McCormick and Pape, 1990; Atherton et al., 2010) synaptic responses.

Several reviews of the functional roles of Cav3 calcium channels and HCN channels have been published (Luthi and McCormick, 1998a; Perez-Reyes, 2003; Robinson and Siegelbaum, 2003; Yunker and McEnery, 2003; Biel et al., 2009; Cueni et al., 2009; Wahl-Schott and Biel, 2009; Cain and Snutch, 2010, 2013; Cheong and Shin, 2013; Weiss and Zamponi, 2013). In the current review, we contrast and compare the effects of a new direct molecular interaction between Cav3 and IKCa channels to that of a synergistic interaction between Cav3 and HCN channels on synaptic processing in cerebellar Purkinje cells and deep cerebellar nuclear (DCN) cells, the postsynaptic target of Purkinje cells. These two examples serve to emphasize how ion channel interactions involving Cav3 channels can result in a wide range of functional effects.

## CEREBELLAR SYNAPTIC INPUTS

**FIGURE 1:**
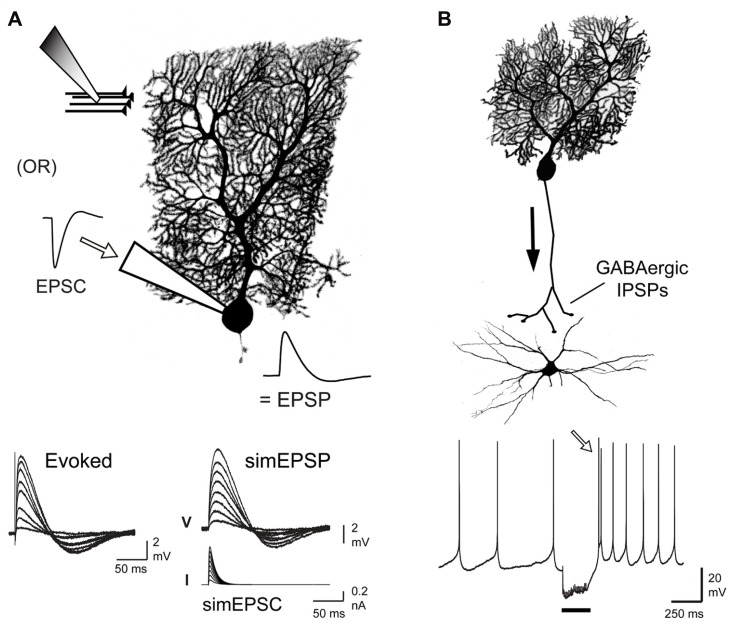
**Comparison of postsynaptic responses modulated by Cav3 channel-mediated calcium influx.** **(A)** Schematic illustrating means of evoking EPSPs through direct stimulation of parallel fiber input or by injecting simulated EPSCs through the electrode at the soma of a Purkinje cell. Traces below compare evoked and simEPSPs over a range of stimulus intensities. **(B)** Schematic of the inhibitory GABAergic input provided by Purkinje cells to DCN cells. Trace below shows a tonically firing DCN cell and a rebound firing response (*open arrow*) following a 100 Hz train of Purkinje cell input (20 pulses, *black bar*). All cells were biocytin filled during whole-cell recordings and labeled by streptavidin-Cy3. Modified from Engbers et al. (2012) and Tadayonnejad et al. (2010).

The cerebellum is a highly organized cortical structure that receives information from the periphery as well as input from descending cortical projections relayed through pontine nuclei in the form of excitatory synaptic inputs. The majority of input arrives as mossy fibers to granule cells that in turn project parallel fibers (PF) to the molecular layer, providing upwards of 150,000 inputs to individual Purkinje cells. Not all of these inputs are active, as long-term depression results in the silencing of up to 85% of the PF-PC synapses (Ekerot and Jorntell, 2001; Isope and Barbour, 2002). However, even accounting for silenced synapses, the Purkinje cell can still receive input from over 20,000 granule cells. Granule cell projections are highly organized in the context of sensory input but even a low rate of spontaneous activity (~1–4 Hz) can lead to an enormous level of background PF activity that Purkinje cells must discriminate from sensory-relevant bursts of input in a small subset of PFs (Chadderton et al., 2004; Rancz et al., 2007; Ekerot and Jorntell, 2008; D’Angelo and De Zeeuw, 2009). Purkinje cells that are excited by granule cells project inhibitory GABAergic output to cells in the DCN, a set of three bilateral nuclei at the base of cerebellum. The neural coding strategies used by DCN cells are still being determined, but extensive work *in vitro* has established that they have the capacity to respond to inhibition with a rebound increase in firing rate (Jahnsen, 1986a; Aizenman and Linden, 1999; Molineux et al., 2006, 2008; Tadayonnejad et al., 2009, 2010; Hoebeek et al., 2010; Sangrey and Jaeger, 2010; Engbers et al., 2011). The extent to which this occurs in response to physiological stimuli has recently been debated (Alvina et al., 2008; Person and Raman, 2012), although some consensus begins to emerge from both *in vivo* and *in vitro* work that DCN cells can exhibit a rebound increase in firing frequency given a significantly large inhibitory input that could arise in relation to the frequency, number, and in particular, synchronous input from Purkinje cells (Aizenman and Linden, 1999; Zhang et al., 2004; Wetmore et al., 2008; Tadayonnejad et al., 2009, 2010; Hoebeek et al., 2010; Bengtsson et al., 2011). Nevertheless, the ionic mechanisms that could facilitate a rebound response are still under investigation. The effects of PF input to Purkinje cells or Purkinje cell input onto DCN cells can be studied in an *in vitro* slice preparation either by directly stimulating synaptic inputs or by simulating synaptic potentials by injecting postsynaptic current waveforms at the soma (**Figure 1**). The current review compares the means by which Cav3 channels can interact with other ion channels to produce markedly different results between the PF-Purkinje cell synapse and the Purkinje-DCN cell synapse.

## THE ROLE OF CAV3 CHANNELS IN SUPPRESSING TEMPORAL SUMMATION

Parallel fibers EPSPs are followed by an afterhyperpolarization (AHP) lasting up to ~200 ms, a striking feature given that these AHPs can be seen to follow even small amplitude EPSPs. Part of this AHP is due to deactivation of *I*_H_ during the depolarizing phase of the EPSP, resulting in a hyperpolarizing voltage overshoot (Angelo et al., 2007). However, we recently showed that the remaining portion of the AHP is due to activation of IKCa channels via Cav3 channel-mediated calcium influx at hyperpolarized potentials (Engbers et al., 2012). This finding improves on our understanding of Cav3 ion channel interactions in three ways: (1) Cav3 channels can activate at much lower voltages than previously thought, (2) small amplitude Cav3-mediated current is sufficient to activate KCa channels, and (3) the functional role of Cav3 inward current can be inverted by association with a K^+^ channel.

**FIGURE 2:**
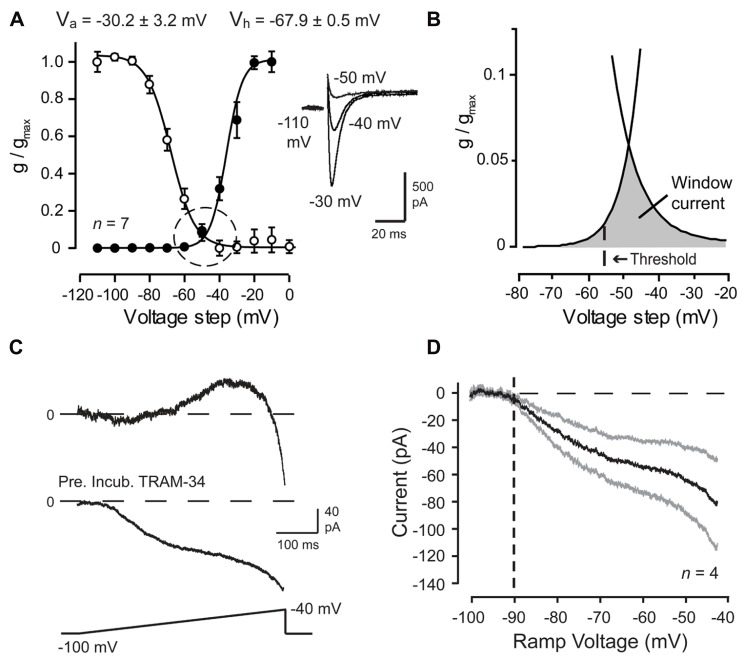
**Cav3 T-type calcium current is evoked over a wide membrane voltage range in Purkinje cells.** **(A,B)** Mean conductance and inactivation plots calculated for whole-cell Cav3 current recorded from P10–12 Purkinje cells. *Inset* shows Cav3 current evoked over relatively negative step commands. **(B)** Expanded image of the fits for activation and inactivation curves from plots in **(A)** (*dashed circle*) showing a wide voltage range for Cav3 window current (*gray*) in relation to spike threshold (*dashed line*). **(C,D)** Whole-cell recordings from mature (P18–30) Purkinje cells evoked by a ramp from -100 mV to -40 mV (500 ms) and Ni^2+^-sensitive currents extracted by applying 300 μM Ni^2+^. Shown are records of sequential inward-outward currents activated in one cell (*top trace*) and in another cell purely inward current when recorded in the presence of the potassium channel blocker TRAM-34 (100 nM) (*bottom trace*). **(D)** Current-voltage plot of the average Ni^2+^-sensitive Cav3 current evoked by a ramp command in the presence of TRAM-34. Inward current exhibits initial activation from a voltage of ~-90 mV (*dashed line*). Standard errors are shown by gray traces. Modified from Engbers et al. (2012).

Cav3 channels are known to be low voltage-activated, with the voltage for initial activation usually identified by the presence of measureable current on conductance plots derived from whole-cell voltage clamp recordings. However, it is important to recognize that activation of channels in the membrane may occur at voltage levels below those necessary to appear under whole-cell recording conditions. In this regard, voltage clamp analyses have established that Cav3 calcium channels exhibit overlap in the activation and inactivation profiles. This overlap establishes a window current that, while small near resting membrane potential (~2% of total available current), is constitutively active and sufficient to raise intracellular calcium concentration and affect neuronal firing (**Figures 2A, B**; Huguenard, 1996; Swensen and Bean, 2003; Perez-Reyes, 2006; Talavera and Nilius, 2006; Dreyfus et al., 2010; Engbers et al., 2012). Careful inspection of the degree of overlap of these curves recorded even under whole cell conditions reveals a Cav3 window current that extends from well below -65 mV all the way to ~-20 mV (**Figure 2B**). These values agree with previous work reporting that a significant proportion of calcium current activated during a sodium spike in Purkinje cells is mediated Cav3 channels (Chemin et al., 2002; Swensen and Bean, 2003). Therefore, LVA Cav3 channels can be a significant source of calcium at subthreshold and suprathreshold voltages, and provide a constitutive inward current even during rest.

Activation curves derived by voltage steps in whole-cell voltage clamp might suggest that no significant activation of Cav3 current will occur at potentials negative to -70 mV. However, in a study of HVA calcium channel-mediated transmitter release, the use of slow voltage ramps revealed Cav2.1 (P/Q-type) channel activation ~20 mV more negative than the activation threshold suggested by macroscopic activation curves (Awatramani et al., 2005). Experimental results had also suggested that Cav3 channels were capable of being activated at potentials as low as -75 mV (Engbers et al., 2012). Indeed, slow ramp commands (-100 to -40 mV) applied to Purkinje cells in whole-cell configuration established initial activation of a Ni^2+^-sensitive inward current at voltages as low as -90 mV (**Figures 2C, D**). These and other measures confirm that Cav3 channel window current can extend over a far wider range than previously anticipated, potentially providing a calcium current that can be activated by even small amplitude PF EPSPs. While this does not suggest that potentials of -90 mV will ever be reached under physiological conditions, it does indicate that Cav3 channels can act as a significant source of calcium influx over the entire subthreshold range and their availability as a functional current should not be discounted based solely on membrane potential in relation to the voltage for inactivation. In fact, Cav3 channels are known to contribute to Purkinje cell activity during tonic firing at a depolarized state (Swensen and Bean, 2003; Engbers et al., 2012). Furthermore, Cav3 activation properties are known to vary between cell types and even be regulated by their association with other structures, such as mGluR1 receptors (Hildebrand et al., 2009).

In addition to the Ni^2+^-sensitive inward current, ramp commands in Purkinje cells revealed a Ni^2+^-sensitive outward current that activated from ~-80 mV with a later onset than the initial Cav3 calcium current (**Figure 2C**), presumably the same current responsible for the PF-associated AHP. This finding indicated at least a functional coupling between Cav3 channels and KCa channel(s) of some identity. BK and SK2 channels are both expressed in Purkinje cells and are known to be activated by calcium influx. A functional coupling and molecular association between HVA calcium and BK channels is well-established and characterized (Raman and Bean, 1999; Swensen and Bean, 2003; Grunnet and Kaufmann, 2004; Womack et al., 2004; Berkefeld et al., 2006; Alvina and Khodakhah, 2008; Berkefeld and Fakler, 2008; Fakler and Adelman, 2008). A functional coupling between BK and Cav3 channels has also been demonstrated in some cells (Smith et al., 2002; Engbers et al., 2013; Rehak et al., 2013). A higher sensitivity of SK channels to intracellular calcium compared to BK channels allows the SK channel family to couple with a wide range of Ca^2+^ sources, including Cav1, Cav2.3, and Cav3 calcium channels, NMDA receptors, and Ca^2+^-permeable nicotinic acetylcholine receptors (Marrion and Tavalin, 1998; Oliver et al., 2000; Wolfart and Roeper, 2002; Ngo-Anh et al., 2005; Yanovsky et al., 2005; Bloodgood and Sabatini, 2007; Cueni et al., 2008; Fakler and Adelman, 2008; Faber, 2010).

**FIGURE 3:**
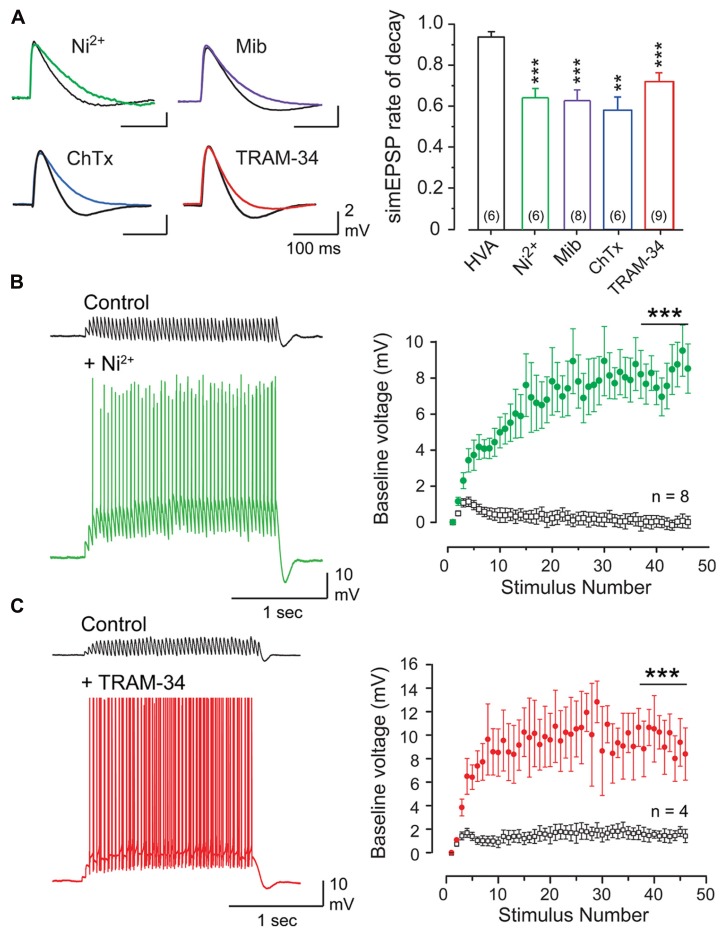
**The parallel fiber EPSP activates a Cav3- and KCa3.** 1-mediated AHP that suppresses temporal summation. **(A)** Superimposed records of simEPSPs in Purkinje cells before and after 100 μM Ni^2+^, 1 μM mibefradil (Mib), 100 nM ChTx, or 100 nM TRAM-34. Bar graphs show mean values of the reduction of simEPSP rate of decay. HVA refers to a cocktail of ω-conotoxin GVIA (1 μM), nifedipine (1 μM), and SNX-482 (200 nM). **(B,C)** Recordings and plots of the mean baseline voltage during 25 Hz trains of parallel fiber-evoked EPSPs before and after applying Ni^2+^ [**(B)**, 100 μM)] or TRAM-34 [**(C)**, 100 nM]. Recordings in **(B,C)** were conducted in 50 μM picrotoxin. Statistical significance tested for last 10 pulses of stimulus trains in **(B,C)** is denoted by bars. Sample values in **(A)** are shown in brackets within bar plots. ^**^p < 0.01, ^***^p < 0.001. Modified from Engbers et al. (2012).

Given the wide range of calcium sources capable of activating BK and SK channels, it was fully expected that one of these KCa channel subtypes would account for the PF-evoked AHP. However, a pharmacological assay established that the PF EPSP-AHP simulated by postsynaptic injection of a PF EPSC waveform (simEPSP) was fully *insensitive* to blockers of SK or BK channels as well as a range of HVA calcium channel blockers (Engbers et al., 2012). Instead the PF EPSP rate of decay and AHP were reduced by low Ni^2+^ and mibefradil, two established blockers of Cav3 current in Purkinje cells (McDonough and Bean, 1998; Isope and Murphy, 2005; **Figure 3A**). The rate of decay of the simEPSP and AHP were also blocked by charybdotoxin (ChTx) or TRAM-34, two blockers of IKCa channels (**Figure 3A**). This was surprising given that IKCa channels were only believed to be expressed in central regions in activated microglia and endothelial cells (Kaushal et al., 2007; Pedarzani and Stocker, 2008). However, applying TRAM-34 during ramp commands effectively blocked all Ni^2+^-sensitive outward current (**Figures 2C, D**). Moreover, a series of protein biochemical tests established that IKCa channels are expressed in Purkinje cells and exhibit an association with Cav3 channels at the molecular level (see Engbers et al., 2012). These results then established the existence of a Cav3-IKCa channel complex, adding to the number of complexes recognized between calcium and potassium channels.

It is interesting to contemplate the pairing of Cav3 and IKCa channels. Cav3 channels have a small single channel conductance, low maximal open probability and exhibit rapid inactivation, resulting in small changes in intracellular calcium during subthreshold voltage changes. Therefore, any K_Ca_ channel depending on Cav3-mediated calcium influx would have to be exquisitely sensitive to calcium concentration. Interestingly, IKCa is the most sensitive of the K_Ca_ channels and exhibits a slow rate of deactivation once activated (Ishii et al., 1997; Joiner et al., 1997). Furthermore, the larger conductance of IKCa channels (20–90 pS) compared to SK channels (10–20 pS) allows small changes in open probability to have a greater effect on membrane voltage. The molecular association between Cav3 and IKCa channels is thus an ideal pairing of signal and transducer, with the K_Ca_ channel being tuned to sense the range of intracellular calcium concentration changes associated with Cav3 influx at the level of a nanodomain.

Given the factors that allow Cav3 calcium influx to activate IKCa channels, this ion channel complex should exhibit activation in the subthreshold region. Repetitive PF stimulation revealed an initial temporal summation of EPSPs over the first few stimuli, but then a marked suppression of summation for the remainder of the stimulus train (**Figures 3B, C**). This result was obtained in the presence or absence of picrotoxin to block GABAergic inhibition (not shown), revealing that suppression of temporal summation did not involve feedforward inhibition activated by PF inputs (Hausser and Clark, 1997; Mittmann et al., 2005). Rather, perfusion of either Ni^2+^ (100 μM) to block Cav3 calcium influx or TRAM-34 (100 nM) to block IKCa channels restored temporal summation to reveal a substantial increase in EPSP peak amplitude and repetitive spike output from Purkinje cells throughout the stimulus train (**Figures 3B, C**). Therefore, when coupled to IKCa channels, the Cav3 channel does not provide a net depolarizing effect, but has its influence effectively inverted by the IKCa channel, resulting in a net hyperpolarizing effect. The close association between these channels further allow a rapid response to subthreshold depolarizations and a pronounced effect on temporal summation of excitatory inputs.

These studies were important in demonstrating that Cav3 calcium current has the capacity to activate at very negative membrane voltages, providing an inward current that could potentially accentuate temporal summation and other membrane depolarizations. However, by forming a complex with IKCa channels, Cav3 calcium influx instead provides a net inhibitory influence over temporal summation. These and other experiments help support the hypothesis that the purpose of a Cav3-IKCa mediated inhibition is to suppress the background level of PF input expected to be transmitted to Purkinje cells (Engbers et al., 2012). In this way Purkinje cells will be able to respond to the high frequency bursts of PF input that convey sensory-relevant information.

## SYNERGISTIC INTERPLAY BETWEEN CAV3 AND HCN CHANNELS BOOSTS A REBOUND RESPONSE

A different interaction between Cav3 and HCN channels becomes apparent in the integration of inhibitory synaptic input to DCN cells. Recordings *in vitro* have primarily focused on large diameter cells that exhibit a characteristic rebound increase in firing rate following a period of membrane hyperpolarization or train of IPSPs evoked by stimulation of Purkinje cell input (Molineux et al., 2008; Tadayonnejad et al., 2009, 2010; Pedroarena, 2010; Sangrey and Jaeger, 2010; Engbers et al., 2011). Transient calcium current through Cav3 channels (*I*_T_) and the inward tail current associated with hyperpolarization-activated HCN channels (*I*_H_) have been proposed to contribute to rebound responses from some of the earliest recordings (Jahnsen, 1986b; McCormick and Pape, 1990; Muri and Knopfel, 1994; Huguenard, 1996). We also found a correlation between the expression of the Cav3.1 calcium channel isoform in DCN cells and the generation of a high frequency Transient Burst phenotype, and for Cav3.3 channels to a form of Weak Burst response (Molineux et al., 2006). For the purpose of this review we will restrict examples to Transient Burst DCN cells.

**FIGURE 4:**
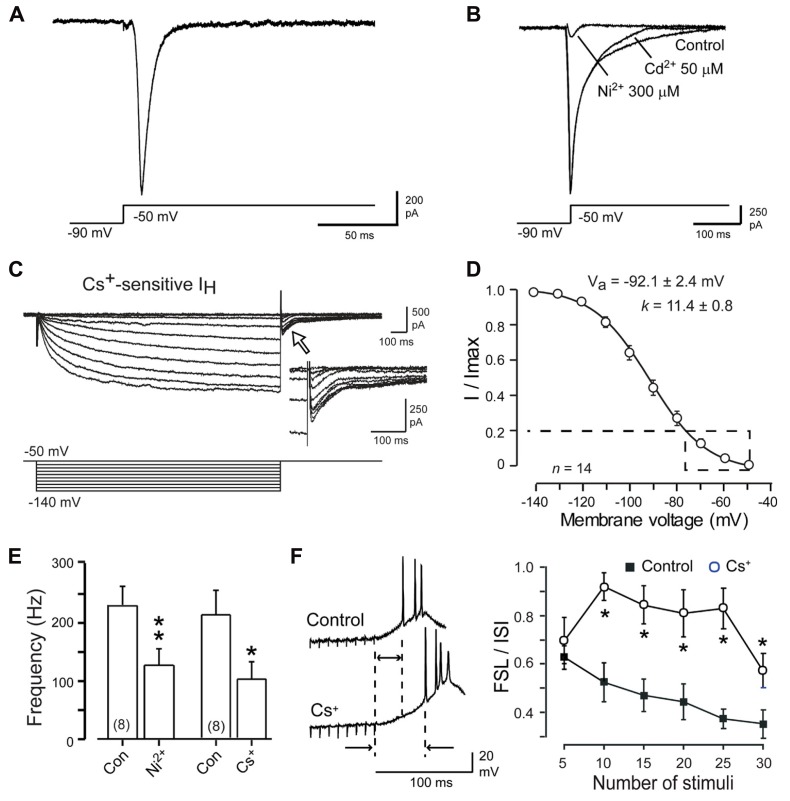
**DCN cells express both *I***_**T**_ and *I*_**H**_. **(A)**, *I*_T_ recorded from a Transient Burst DCN cell under whole-cell conditions at physiological temperature for a single step to -50 mV. **(B)** *I*_T_ recorded in a separate cell reveals no effects of applying Cd^2+^ but nearly full block by Ni^2+^. **(C)** *I*_H_ recorded from a DCN cell at physiological temperature and identified as Cs^+^-sensitive current. An inward tail current is evident following the end of step commands (*inset, open arrow*). **(D)** Mean conductance plot for Cs^+^-sensitive *I*_H_. *Dashed line* and *box* denotes the physiological range of membrane voltage traversed by IPSPs. **(E)** Recordings and mean bar plots of rebound responses in DCN cells evoked by a hyperpolarizing current pulse reveal a decrease in burst frequency after blocking either Cav3 (Ni^2+^) or HCN (Cs^+^) channels. **(F)** Blocking *I*_H_ with Cs^+^ reveals an increase in FSL during the rebound response evoked by a train of Purkinje cell IPSPs that increases in a graded fashion with the number of stimuli. ^*^p < 0.05, ^**^p < 0.01. Modified from Molineux et al. (2008) and Engbers et al. (2011).

*I*_T_ recorded under steady-state whole-cell voltage clamp conditions is shown in **Figure 4**, where a step from a negative holding potential to -50 mV (a value just below spike threshold) reveals a large amplitude calcium current. The correspondence of this current to Cav3 channels is supported by an insensitivity to the general HVA calcium channel blocker Cd^2+^ (50 μM) but full block by 300 μM Ni^2+^ (**Figures 4A, B**). *I*_H_ recorded under whole cell conditions and isolated as Cs^+^-sensitive current is evident as a slowly activating and non-inactivating inward current that activates at physiological temperatures negative to -50 mV (**Figures 4C, D**). Of most apparent relevance to a rebound response is a prolonged inward *I*_H_ tail current (~500 ms) that follows the end of a negative command pulse (Engbers et al., 2011). This is important in that a prolonged *I*_H_ deactivation will be expected to generate a depolarization with potential influence on the rebound response (Aizenman and Linden, 1999; Engbers et al., 2011; Steuber et al., 2011). Indeed, perfusion of either a low concentration of Ni^2+^ (300 μM) or Cs^+^ (2 mM) under current clamp conditions reduced the frequency of the rebound response (**Figure 4E**), results consistent with a depolarizing influence for both *I*_T_ and *I*_H_. In fact, the ability for calcium channel blockers to reduce the rebound response has been reported using voltage clamp step commands (Molineux et al., 2006, 2008), calcium imaging experiments (Muri and Knopfel, 1994; Zhang et al., 2004; Zheng and Raman, 2009; Schneider et al., 2013), and recently more selective *I*_T_ blockers (Alvina et al., 2009; Boehme et al., 2011). Blocking *I*_H_ with external Cs^+^ uncovered an additional influence of *I*_H_ in controlling the first spike latency (FSL) to the burst response, an effect that was graded with the stimulus (**Figure 4F**; Sangrey and Jaeger, 2010; Tadayonnejad et al., 2010). Thus, the greater the number of stimuli (and the longer the hyperpolarization) the shorter the FSL when expressed as a ratio to the control interspike interval (ISI). Similar results on FSL/ISI were not obtained by perfusing Cav3 channel blockers (not shown; Engbers et al., 2011).

**FIGURE 5:**
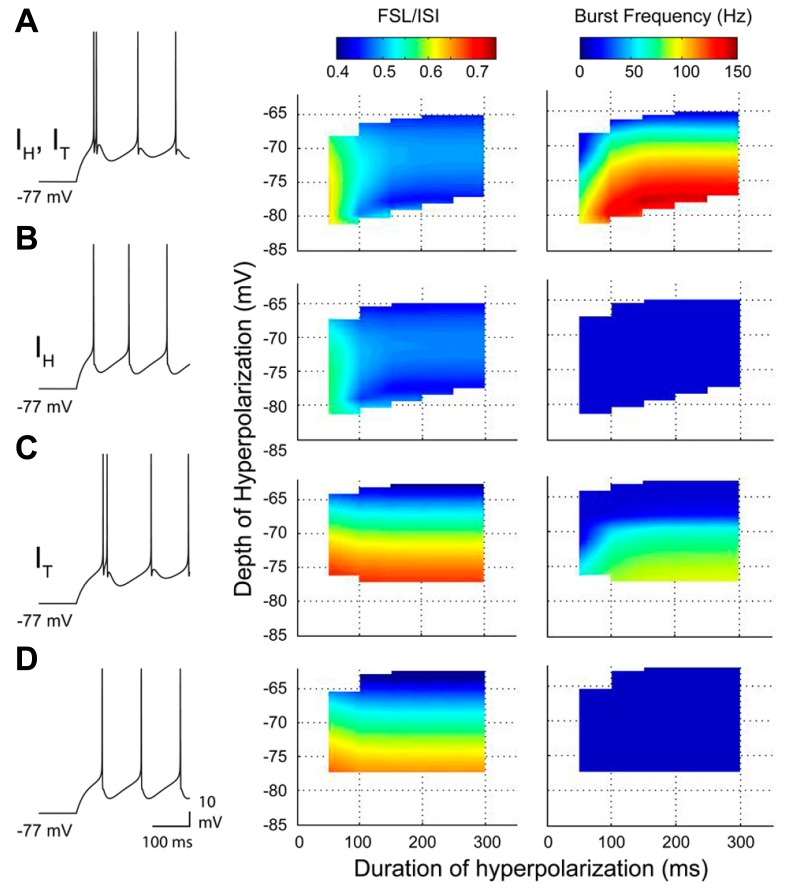
**A reduced model of the roles for *I***_**T**_ and *I*_**H**_ in rebound response frequency and FSL. **(A–D)**, Output from a two compartment model containing combinations of *I*_T_ and/or *I*_H_, with representative spike output following a 300 ms hyperpolarizing step to -77 mV from a resting potential of ~-60 mV (10 Hz baseline firing) (*left*). Changes in frequency and FSL/ISI are plotted in three dimensions in relation to the frequency and ISI present at a resting membrane potential of ~-60 mV. Note a decrease in FSL/ISI and increase in frequency with longer durations or larger amplitude hyperpolarizations when both *I*_T_ and *I*_H_ are present **(A)**. Removing *I*_T_ dramatically abolishes all rebound responses **(B)**, while removing *I*_H_ reverses the FSL/ISI relationship and reduces rebound frequency **(C)**. Removing both *I*_T_ and *I*_H_ blocks the shifts in both FSL/ISI and spike frequency found when both are present **(D)**.

The most direct interpretation of these results is that both Cav3 and HCN channels contribute a membrane depolarization that directly increases rebound spike frequency and reduces the FSL. To more carefully compare the roles for *I*_T_ and *I*_H_ in either aspect of a rebound, we used available data on these currents (**Figures 2 and 4**) to construct a two compartment reduced model based on Hodgkin–Huxley formalism (for details, see Engbers et al., 2011). Importantly, this model proved capable of reproducing the stimulus-dependent decrease in FSL/ISI and increase in spike frequency of the rebound response (**Figure 5A**). We note that the frequency response here was measured over only the first 100 ms of the rebound, and does not account for the longer duration depolarizations that reflect contributions by HVA calcium channels or *I*_NaP_ (Zheng and Raman, 2009; Sangrey and Jaeger, 2010; Steuber et al., 2011). Comparing the output of the model upon removing *I*_T_ or *I*_H_ was highly instructive in first showing that if only *I*_H_ was present the model preserved the inverse relationship between FSL/ISI and hyperpolarization strength (greater depth and longer duration) but also exhibited a significant decrease in rebound frequency (**Figure 5B**). If only *I*_T_ was present, the FSL/ISI relationship was reversed, with longer hyperpolarizations resulting in a longer FSL/ISI ratio (**Figure 5C**). However, the rebound response was only present when the model contained *I*_T_ (**Figures 5A, C**). Removing both *I*_T_ and *I*_H_ caused the FSL/ISI relationship to become directly proportional to the depth and duration of hyperpolarization, as would be expected without active currents in the subthreshold range, and abolished the rebound response (**Figure 5D**).

These results emphasize that *I*_H_ is the primary determinant of the FSL of a rebound response, while *I*_T_ by itself failed to reproduce the normal FSL response. In addition, the large reduction in rebound frequency when *I*_H_ was removed revealed that *I*_H_ has a key role in controlling the frequency of a rebound response. The most straightforward explanation for this would be that *I*_H_ increases rebound frequency by way of a direct depolarization of the membrane following hyperpolarization. However, further examination revealed that the contribution by *I*_H_ to rebound frequency could instead be accounted for by a synergistic effect of *I*_H_ on *I*_T_, revealing an *indirect* interaction between HCN and Cav3 channels.

**FIGURE 6:**
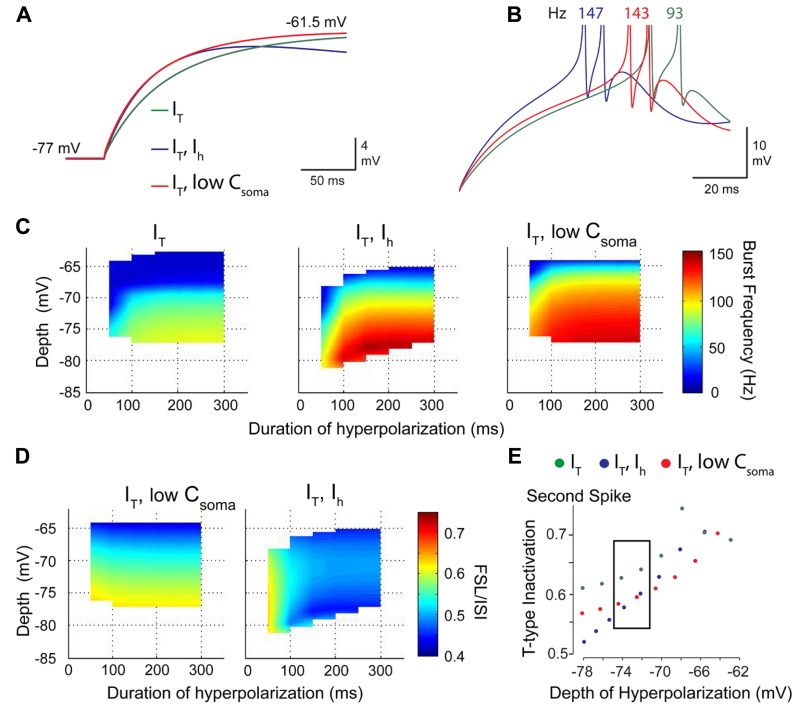
***I***_**H**_ acts to increase the role of *I*_**T**_ in determining rebound burst frequency by modifying the membrane time constant. **(A)** Expanded image of membrane charging profiles during repolarization from -77 mV. Superimposed traces are for models containing *I*_T_ and *I*_H_ (*I*_T_–*I*_H_), *I*_T_ alone, and a model with only *I*_T_ in which membrane capacitance was lowered (*red trace*) to match the time constant for a model containing *I*_H_ (*purple trace*). **(B)** In the absence of *I*_H_, a decrease in capacitance increases burst frequency and decreases FSL (*red trace*) when compared to a model with *I*_T_ alone (*green trace*). **(C)** Three dimensional colour plots comparing the response of models containing *I*_T_, *I*_T_–*I*_H_, and *I*_T_ with a low somatic membrane capacitance to simulate the effect of *I*_H_. Note that the *I*_T_-_LowC_ model reaches the same peak frequency of 150 Hz as the *I*_T_–*I*_H_ model, indicating that a reduction in time constant increases burst frequency. **(D)** A comparison of models of *I*_T_-_LowC_ to *I*_T_–*I*_H_ indicate that the voltage-dependence and kinetics of *I*_H_ determine the normal voltage-FSL relationship. **(E)** A comparison of the amount of *I*_T_ inactivation during the rebound between the three models shows that incorporating low C or *I*_H_ reduces the extent of *I*_T_ inactivation compared to a model of *I*_T_ alone. Modified from Engbers et al. (2011).

Recognition of an *I*_T_–*I*_H_ interaction was gained when considering a well-established role for HCN channels in regulating resting membrane conductance. In hippocampal cells, *I*_H_ has been shown to account for a large percentage of the resting conductance and acts to normalize dendritic EPSPs (Magee, 1999). In Purkinje cells, basal activation of *I*_H_ at resting membrane potentials shortens the width of PF EPSPs (Angelo et al., 2007) and reduces the bistable range (Williams et al., 2002; Fernandez et al., 2007). To determine if *I*_H_ exerted its role through a direct depolarization or by modifying the membrane conductance, we compared charging curves of the three models for *I*_T_ alone, *I*_T_–*I*_H_, and when *I*_T_ was expressed alone and membrane capacitance adjusted in the somatic compartment (*I*_T_-C; **Figure 6A**). This test showed that the model of *I*_T_ alone had a slower rate of charging compared to *I*_T_–*I*_H_ or *I*_T_-C, but that a model of *I*_T_ could be adjusted to the same time constant when model capacitance was increased as a substitute for *I*_H_ (**Figure 6A**). When compared in current clamp, it was apparent that spikes discharged at a shorter latency for either the *I*_T_–*I*_H_ model or *I*_T_-C than for *I*_T_ alone (**Figure 6B**). These results were borne out when comparing three dimensional plots of the rebound burst frequency for different levels and durations of hyperpolarization, where the profile of burst frequency for the *I*_T_-C strongly resembled that of *I*_T_–*I*_H_ (**Figure 6C**). In contrast, adjusting the capacitance in a model with just *I*_T_ could not replicate the FSL/ISI relationship generated by a model of *I*_T_–*I*_H_ (**Figure 6D**), indicating that the voltage dependence and kinetics of *I*_H_ determine the FSL/ISI relationship. Finally, a comparison of the degree of *I*_T_ inactivation for different depths of hyperpolarization revealed that without *I*_H_ the membrane potential returned to rest at the end of the stimulus slowly enough to induce greater *I*_T_ inactivation, and thus less ability to contribute to a rebound response (**Figure 6E**). Thus, a normally slow rate of return of membrane potential at the end of a stimulus is rescued by virtue of the effects of *I*_H_ on the membrane time constant, an effect that can be mimicked by lowering cell capacitance.

**FIGURE 7:**
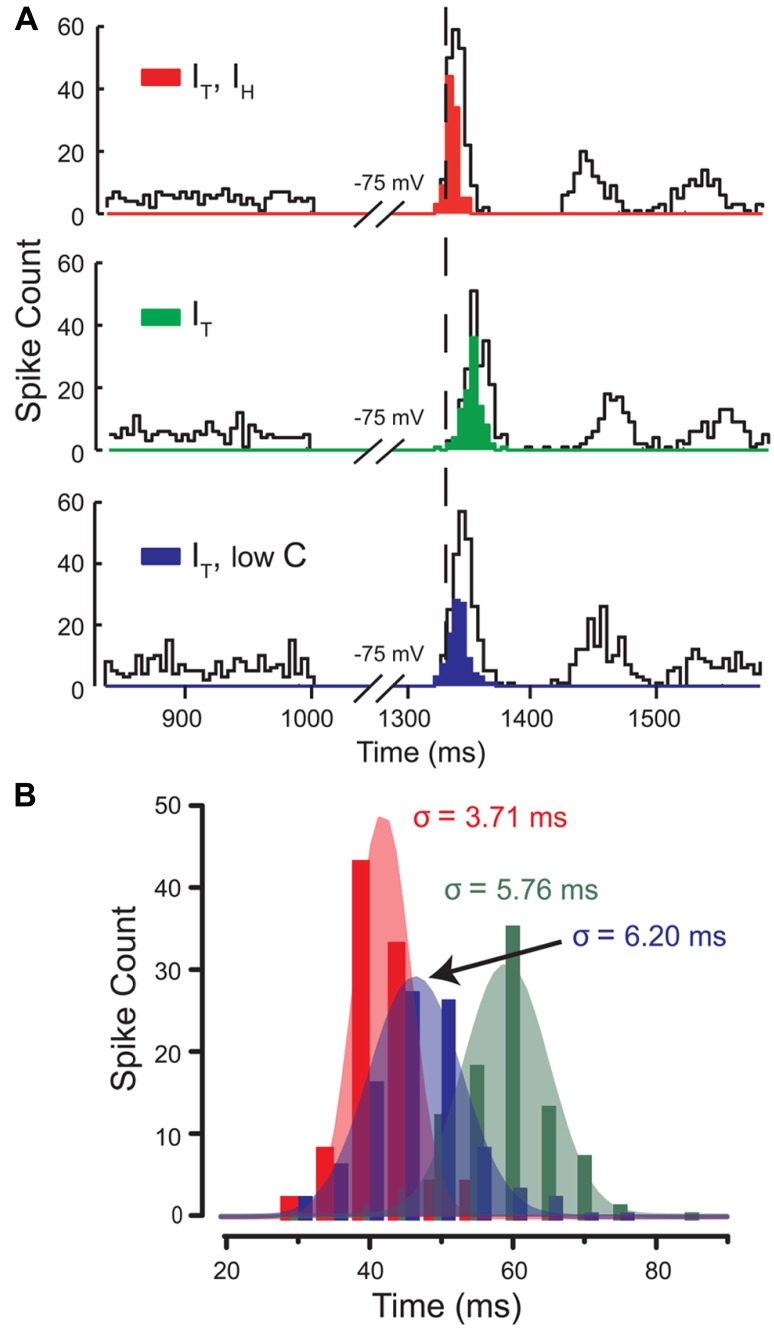
***I***_**H**_ controls the precision of the first spike following a hyperpolarization. **(A)** Histograms of spike count in three different models for 100 simulations with stochastic excitatory and inhibitory synaptic conductances. *Open bins* denote the total spike count, while *filled bins* denote the first spike following release from inhibition of each of the 100 simulations in the population of rebound responses. The mean latency (relative timing) and standard deviation of FSL (spike precision) between three models indicate that the effects of *I*_H_ governs both the timing and precision of first spikes in the rebound. **(B)** Gaussian fits of the population of first spikes for each model in **(A)** shows that when *I*_H_ is present (*red*), FSL falls over a narrow distribution and with small standard deviation (increased spike precision) compared to models lacking *I*_H_ (*green*) or with lowC (*blue*). Modified from Engbers et al. (2011).

A second important parameter of FSL is the degree of precision of the first spike, as measured by the standard deviation of the latency of jitter in a population of spike discharges. To test the contribution of *I*_H_ and *I*_T_ to this parameter, we injected the models with stochastic excitatory and inhibitory synaptic conductances and injected current to hyperpolarize the model to -75 mV for 300 ms (**Figures 7A, B**). The standard deviation of the latency of discharge of the first spike for each of the different models was used as a measure of spike precision. In a model of *I*_T_–*I*_H_ the latency to first spike displayed a high degree of precision or minimal standard deviation (**Figures 7A, B**). By comparison, the model with *I*_T_ alone presented a much greater degree of variation in FSL, the majority of which were at longer latencies than for the model of *I*_T_–*I*_H_ (**Figures 7A, B**). Here, a model of *I*_T_ alone with an adjustment of membrane capacitance could not restore spike precision despite a decrease in latency (**Figures 7A, B**). This suggests that the direct depolarizing effect of *I*_H_ following hyperpolarization affects the precision of the first spike. These data were important in showing that *I*_H_ modifies the actions of *I*_T_ both by decreasing latency and increasing precision of first spike discharge, but through different mechanisms.

## CONCLUDING REMARKS

The processing of synaptic input requires the activation of ion channels in a low voltage range in order to modify subthreshold potentials. Cav3 calcium channels are low voltage-activated and are unique among calcium channels in exhibiting fast activation and voltage-dependent inactivation, leading to only a transient current when activated. These characteristics suggest that T-type current would have rapid but only short lasting effects on membrane excitability given the extent to which membrane depolarizations will inactivate the channels. In addition, unlike HVA calcium channels, Cav3 channels were long believed not to associate with any particular potassium or ligand-gated channel. However, in recent years this outlook has changed with identification of functional and even molecular associations between Cav3 calcium and other channels. Some of the first reports revealed that Cav3 channels can at least functionally couple to activation of BK and SK potassium channels (Smith et al., 2002; Cueni et al., 2008). More recently Cav3 channels were shown to associate at the molecular level with BK and IKCa channels and even Kv4 A-type potassium channels (Anderson et al., 2010a, b; Engbers et al., 2012, 2013; Rehak et al., 2013). These associations prove capable of greatly expanding the role for Cav3 calcium channels in processing input signals and modifying cell output.

A critical determinant of Cav3 channel activity is the extent of window current defined by the overlap of activation and inactivation curves. We now recognize that the extent of this overlap can be far greater than expected and to extend well above spike threshold, allowing evoked spikes to incorporate a significant component of T-type calcium current (Chemin et al., 2002; Swensen and Bean, 2003). More recently it was shown that activation of Cav3 calcium current can be detected electrophysiologically from potentials as low as -90 mV in cerebellar Purkinje cells (Engbers et al., 2012) and -70 mV in MVN neurons (Rehak et al., 2013). This is important in indicating that inward T-type current can be activated well within the range of subthreshold depolarizations or hyperpolarizations. While activation of inward current should provide a depolarizing drive, the influx of calcium can secondarily alter the net effect of calcium influx in ways we are only beginning to appreciate. In addition, other channels not known to physically associate with Cav3 channels but which affect voltage transitions can secondarily affect the functional role of Cav3 channels. The current review focused on two recently identified interactions between Cav3 and IKCa channels and Cav3 and HCN channels as a means of highlighting two very different forms of interactions involving Cav3 channels.

## Cav3-IKCa MODULATION OF TEMPORAL SUMMATION

In cerebellar Purkinje cells a direct association between Cav3 and IKCa channels enables the activation of outward potassium current well into the subthreshold voltage range. Thus, even small amplitude PF EPSPs are capable of activating Cav3 channels and subsequently IKCa outward current to control the rate of decay of the EPSP and a subsequent AHP. The effect this has on temporal summation is dramatic in that only the first few stimuli of a train of EPSPs prove capable of summating, with marked suppression of any further summation even during a long stimulus train. This interaction is important in acting as a high pass filter to reduce the inherent level of excitability of Purkinje cells that would result from the constant barrage of even spontaneous PF EPSPs. Instead, only high frequency bursts of PF input representative of sensory input can exceed the barrier put in place by the Cav3-IKCa association to produce spike output (Engbers et al., 2012). The effects of the Cav3-IKCa complex can be compared to hippocampal and cortical pyramidal cells, where Cav3 channels activated during EPSPs act to amplify EPSP amplitude (Markram and Sakmann, 1994; Magee and Johnston, 1995; Gillessen and Alzheimer, 1997; Urban et al., 1998). In the case of the Cav3-IKCa complex, however, trains of EPSPs are suppressed and excitability is reduced. Similar results have been seen for BK channel activation in MVN neurons, where activation of BK channels by Cav3 calcium influx reduces the gain of spike output, thus reducing cell excitability (du Lac and Lisberger, 1995; Smith et al., 2002; Rehak et al., 2013). Therefore, Cav3 channels can play either an excitatory or a net *inhibitory* role in signal processing depending on the ion channels with which they interact.

## HCN CHANNELS INDIRECTLY MODIFY Cav3 FUNCTIONAL INFLUENCE

Both *I*_H_ and *I*_T_ act within the subthreshold range and often play complementary roles. Early recordings in thalamocortical relay neurons demonstrated that an interplay of *I*_H_ and *I*_T_ generates oscillatory burst output (McCormick and Pape, 1990). In DCN neurons, *I*_H_ and *I*_T_ interact to play distinct yet complementary roles. The direct depolarizing effects of *I*_H_ performs two functions. The first is to drive a graded reduction in FSL as hyperpolarizations are increased, creating an inverse relationship between the strength of the hyperpolarization and the time to burst output (Sangrey and Jaeger, 2010; Engbers et al., 2011). Increased activation of *I*_H_ during hyperpolarization also increases the precision of the rebound burst, improving temporal fidelity. However, activation of *I*_H_ also improves the efficacy of *I*_T_. By reducing the membrane time constant, *I*_H_ decreases the inactivation of *I*_T_ during repolarization, resulting in more Ca^2+^ influx and higher burst frequency. *I*_H_ thus acts synergistically with *I*_T_ to increase rebound frequency by at least 60% of the total response. As short-term facilitation and depression are known to be highly frequency-dependent (Dittman et al., 2000), the ability for *I*_H_ to increase the range of frequencies generated during rebound responses (compare **Figures 5A, C**) could have important physiological consequences at downstream postsynaptic targets of DCN cells (Babalyan and Fanardzhyan, 1991).

## LEVEL OF INTERACTION BETWEEN Cav3 and IKCa/HCN CHANNELS

It is clear that very different outcomes can arise from the expression of Cav3 calcium channels when their conductance or actions on the membrane potential are combined with IKCa or HCN channels. It would thus seem advantageous to employ mechanisms that could coexpress, colocalize, or otherwise ensure the pairing of these channels to mediate their effects in a given cell if not on a regional and subcellular basis. It is interesting to compare our current state of knowledge on the nature of interactions between Cav3 and either IKCa or HCN channels in terms of distribution and mechanisms of co-localization.

All protein biochemistry conducted thus far on the Cav3-IKCa complex has focused on the Cav3.2 channel isoform (Engbers et al., 2012), one of three known members of this family (Cav3.1, Cav3.2, Cav3.3; Perez-Reyes et al., 1998). Western blot analysis indicated coimmunoprecipitation between Cav3.2 and IKCa proteins from cerebellar lysates. This association also appears to be specific to the low voltage Cav3.2 isoform in finding no coimmunoprecipitation between IKCa protein and Cav2.1 (P/Q-type) channels from cerebellar lysates (Engbers et al., 2012), one of the principle HVA calcium channels expressed in Purkinje cells. The physiological interaction between Cav3 calcium influx and IKCa activation in Purkinje cells is also inferred to reflect activity of the Cav3.2 isoform given the ability to block the PF-evoked AHP with 100 μM Ni^2+^, a dose that preferentially blocks Cav3.2 (Zamponi et al., 1996; Lee et al., 1999). Dual immunolabel experiments also revealed a remarkable correspondence in colocalization of both Cav3.2 and IKCa protein to the cell body region and specific patches of proximal dendritic membrane of Purkinje cells. A close proximity of these channels was further indicated by the ability of internal BAPTA, but not EGTA, to block a TRAM-34-sensitive outward current evoked in outside-out recordings from Purkinje cells. This test is argued to support an interaction between channels that function within a calcium nanodomain (<50 nM distance; Berkefeld and Fakler, 2008; Fakler and Adelman, 2008). Together these data indicate that Cav3 and IKCa channels can be associated as a physical complex, although the specific sites for interaction between the subunits have not been reported thus far. We also do not know if there are mechanisms in place to establish this association at the level of the Golgi complex or if the channels are translocated to the membrane (or endocytosed) in any coordinated fashion. This is particularly the case for IKCa channels that can be highly regulated in terms of stimulus-induced transcription, translation, and translocation to and from the plasma membrane (Ghanshani et al., 2000; Toyama et al., 2008; Wulff and Castle, 2010; Bouhy et al., 2011; Schwab et al., 2012). The current evidence only identifies a coimmunoprecipitation between Cav3.2 and IKCa from brain lysates, and a functional interaction at the plasma membrane level with minimal physical separation. Further work will thus be required to establish where and how the Cav3.2-IKCa interaction is formed and the extent to which it might influence functions of the complex.

The ability to identify a Cav3.2-IKCa interaction also does not guarantee that all cells expressing Cav3.2 and IKCa proteins will form these ion channel complexes. Precedence for this can found in previous work showing that activation of BK channels by calcium influx through N-type channels can be detected in close association in a given cell (as recognized by paired activation of N-type and BK channels; Marrion and Tavalin, 1998; Berkefeld et al., 2006; Loane et al., 2007; Berkefeld and Fakler, 2008). L-type calcium channels can complex with SK potassium channels and still be recorded in adjacent membrane in isolation (Marrion and Tavalin, 1998). These factors must therefore be considered when one attempts to predict where a Cav3-IKCa complex might be active in cerebellum or other brain regions. The reported expression pattern of Cav3 channels differs according to available reports on mRNA expression through *in situ* hybridization or protein distribution by immunocytochemistry. Indeed, the most thorough *in situ* hybridization work predicted that Purkinje cells express Cav3.1 mRNA (Craig et al., 1999). In contrast, immunolocalization suggests that all three isoforms are expressed in Purkinje cells, but differentially across the soma-dendritic axis (Craig et al., 1999; McKay et al., 2006; Molineux et al., 2006; Hildebrand et al., 2009). The Cav3.2 isoform was primarily localized to the somatic region and restricted segments of the primary trunk of apical dendrites, and a lower detectable level on some secondary branches. As indicated above, this distribution proves to be extremely well matched to that of IKCa immunolabel in these cells (Engbers et al., 2012). However, attempting to generalize or predict what other cells might use this complex physiologically is difficult at this time given that the single report of IKCa channels in Purkinje cells represents the first demonstration of this channel type in any central neuron. Comparisons between the pattern of Cav3.2 and IKCa protein in other cells thus awaits a study on the localization of IKCa channels in other brain regions, at which time tentative predictions could be made as to how generalized this interaction might be in controlling cell output.

Much less is know of any potential relationship between Cav3.2 and HCN channels in DCN cells. *In situ* hybridization suggested that the primary mRNA expressed in DCN cells is for Cav3.1. As indicated, a detailed comparison of Cav3 channel immunolabel and rebound burst phenotypes in DCN cells found evidence for a correlation between the expression of Cav3.1 immunolabel and Transient Burst neurons, and Cav3.3 to a Weak Burst phenotype. While Cav3.2 immunolabel could be detected in some group of neurons in the DCN, none of the Transient or Weak Burst cells examined in that study tested positive for this Cav3 isoform. DCN cells have been shown to express immunolabel (Notomi and Shigemoto, 2004) and mRNA (Monteggia et al., 2000; Santoro et al., 2000) for at least HCN1, HCN2, and HCN4 isoforms. However, no comparisons have been made between the pattern of Cav3 and HCN channel labeling to detect any potential colocalization.

Interestingly, at least a functional coupling has been detected between calcium influx and HCN channels in some systems. Thus, calcium influx has been found to augment *I*_H_ in both sino-atrial node cells (Hirano and Hagiwara, 1989) and thalamocortical cells (Luthi and McCormick, 1998b, 1999a, b) by right shifting the voltage-dependence for *I*_H_ activation. In thalamocortical cells the increase in *I*_H_ promotes a slow depolarization of seconds duration that depends on preceding calcium influx from rebound calcium spikes, a response attributed primarily to T-type calcium channels. The increase in *I*_H_ then decreases input resistance and the magnitude of calcium spikes, slowing if not blocking the generation of oscillatory activity (Luthi and McCormick, 1998b). An *I*_H_-dependent process has also been shown to generate a slow depolarization that depends on prior membrane hyperpolarizations in a subset (23%) of cortical neurons, although the calcium dependence of this process was not definitively established (Winograd et al., 2008). Also, this effect was not found in all cortical cell types including those that otherwise expressed *I*_H_, indicating that calcium-dependent regulation of *I*_H_ is not a property of all cells (Winograd et al., 2008).

The calcium dependent increase of *I*_H_ in thalamocortical cells could be blocked by external application of 2–5 mM Ni^2+^ or internal perfusion of BAPTA or EGTA (Budde et al., 1997; Luthi and McCormick, 1998b). While the effects of Ni^2+^ were interpreted as reflecting a block of T-type calcium influx, this concentration is not selective enough to provide strong support for a discrete action on Cav3 channels (Zamponi et al., 1996). The best evidence of a role for T-type channels is the dependence of *I*_H_ augmentation in thalamocortical cells on the generation of rebound calcium spikes (Luthi and McCormick, 1998b). Calcium-dependent regulation of *I*_H_ in these cells was also concluded to be indirect through a calcium-dependent increase of PKA that modifies these cyclic nucleotide-dependent channels (Luthi and McCormick, 1999b). Therefore, at this time, there is evidence that calcium influx can modify *I*_H_ in a subset of neurons, but a direct interaction, like that shown for Cav3 and IKCa channels, has not been demonstrated, with no further work carried out in DCN cells.

It is interesting to note that in cortical layer III pyramidal cells, HCN1 channels colocalize with Cav3.2 channels in the presynaptic terminals of glutamatergic inputs, where they act to increase Cav3 inactivation by depolarizing the terminal (Huang et al., 2011). Similar effects of *I*_H_ increasing *I*_T_ inactivation have been reported for dendrites of hippocampal CA1 pyramidal neurons (Tsay et al., 2007). However, these effects are different from the results summarized here for DCN cells in which the effect of *I*_H_ on membrane time constant instead decreases Cav3 inactivation (**Figure 6**). Thus, at this time we have no reason to suspect that the interplay between Cav3- or HCN-mediated components of the rebound response in DCN cells reflects anything more than coexpressing these channels in isolation in the membrane, with concomitant effects on the membrane time constant and degree of Cav3 channel inactivation.

In summary, work in this area is converging to a growing realization that Cav3 calcium channels do not operate in isolation as long believed, but in fact can interact with other ion channels or receptors indirectly or as functional channel complexes to determine the final influence on membrane excitability. All of this work emphasizes the need for a detailed knowledge of the biophysical properties of ion channels, but also underscores the fact that their functional role depends on what other ion channels they interact with in the membrane simply through coexpression or by direct interaction in a growing number of ion channel complexes.

## Conflict of Interest Statement

The authors declare that the research was conducted in the absence of any commercial or financial relationships that could be construed as a potential conflict of interest.

## AUTHOR CONTRIBUTIONS

Ray W. Turner and Gerald W. Zamponi received grant support for the study; Jordan D. T. Engbers, Dustin Anderson, and Ray W. Turner designed the experiments; Jordan D. T. Engbers and Dustin Anderson conducted the experiments and analyzed the data; Ray W. Turner and Jordan D. T. Engbers wrote the manuscript.
